# Removal of Chromium (VI) by a Magnetic Nanoscale Zerovalent Iron–Assisted Chicken Manure-Derived Biochar: Adsorption Behavior and Synergetic Mechanism

**DOI:** 10.3389/fbioe.2022.935525

**Published:** 2022-07-06

**Authors:** Shengqiong Fang, Xiaoyi Huang, Shuangling Xie, Jiale Du, Jianlong Zhu, Kai Wang, Qinglin Zhuang, Xuan Huang

**Affiliations:** ^1^ College of Environment and Safety Engineering, Fuzhou University, Fuzhou, China; ^2^ Jiangsu DDBS Environmental Remediation Co., Ltd., Nanjing, China

**Keywords:** Cr(VI), biochar, nanoscale zerovalent iron, adsorption, reduction

## Abstract

Using chicken manure as raw material to prepare activated carbon as a dispersant, a novel biochar-loaded nano-zerovalent iron composite (nZVI@CMBC) was developed and applied to remove hexavalent chromium, i.e., Cr(VI), in wastewater. The dispersion of nano-zerovalent iron (nZVI) particles on the surface of chicken manure–derived biochar (CMBC) successfully inhibited the aggregation of magnetic iron particles and effectively reduced the size of nZVI particles. The results demonstrated that under acidic conditions, the removal efficiency of Cr(VI) by the nZVI@CMBC composite could reach 124.12 mg g^−1^. The pseudosecond-order kinetic model had a good agreement with the adsorption kinetics of the nZVI@CMBC composite, implying that the adsorption of Cr(VI) is based on the multi-layer chemical adsorption. Therefore, this study provides a new clue and strategy for removing Cr(VI) in wastewater.

## Graphical Abstract

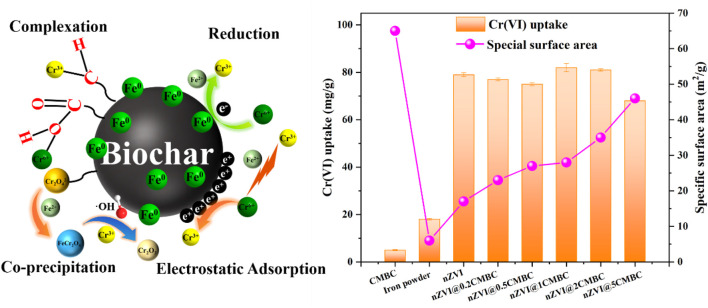

## 1 Introduction

Chromium applications are often involved in electroplating, dyeing, leather, metal pickling, and chromate industries (Amalraj et al., 2016; Lin et al., 2016; Ahmed et al., 2017; Ye J. et al., 2020; Ye W. et al., 2020; Lin et al., 2021). According to the previous reports, Chinese electroplating industries releases about four billion tons of wastewater containing chromium every year, which has caused serious environmental pollution problems (Chen et al., 2015). As a major contributor to chromium pollution, Cr(VI) has relatively high toxicity and carcinogenicity, attracting extensive concentration from environmental researchers (Han et al., 2016). According to the drinking water quality regulations established by the WHO, the concentration of Cr(VI) in drinking water must not exceed 0.05 mg L^−1^ (Atieh et al., 2010; Bhaumik et al., 2012). Therefore, it is urgent to remediate the pollution of Cr(VI), update drinking water standards, and develop high-efficiency treatment technologies (Chen et al., 2015).

As a nanoscale material, zero-valent iron (nZVI) has received widespread attention and is applied in treating wastewater containing heavy metals (Atieh et al., 2010; Debnath et al., 2015). Compared with flocculation, precipitation, membrane separation, and ion exchange, the nZVI oxidation treatment method has unique superiority, such as a simple process, low solid residue, easy separation, and low-cost (Gao and Liu 2016). As the previous literature reported (Hussain et al., 2015), nZVI can minimize the toxicity of Cr(VI) by first absorbing Cr(VI) and then reducing it to Cr(III). For example, Zhu et al. prepared nano zero-valent iron/nickel bimetallic particles to remediate Cr(VI) with the removal rate of 99.36% within 20 min (Zhu et al., 2017; Siying et al., 2018; Zhu et al., 2021). However, due to the high surface energy and magnetic force, nZVI particles are conducive to strong aggregation in micro-scale or large-scale particles (Jalilzadeh and Şenel 2016). To overcome the aforementioned questions, researchers loaded nZVI on the porous materials, such as carbon material and clay (sepiolite, bentonite, or kaolin), to prevent the aggregation and enhance the reduction capacity and utilization of nZVI (Jiang et al., 2012; Karthik and Meenakshi 2015; Ye et al., 2019).

Biochar, as a typical carbon material, has superior performance such as a high specific surface area, little bulk density, high stability, low cost, and fine adsorption performance (Lin et al., 2011; Li et al., 2013; Khan et al., 2016). Based on these advantages, the loading of nZVI onto biochar gradually becomes a research hotspot. According to the previous literature, the biochar-nZVI composite material has a higher specific surface area with more oxygen-containing functional groups (Ruotolo and Gubulin 2011). However, biochar-nZVI composites show the disadvantage of high cost and poor adsorption capacity. For example, Zhang et al. (2019) prepared expensive nZVI-loaded biochar with a Cr(VI) adsorption capacity of 104.4 mg g^−1^. Therefore, it is important to explore new biochar as a dispersant to stabilize nZVI. With the promotion of mechanized and intensive equipment, the aquaculture industry is unprecedentedly developed, resulting in a large amount of animal manure (Liu et al., 2013), and the annual output of chicken manure has exceeded 126 million tons (Lv et al., 2013). Although the chicken manure can be utilized as fertilizer for plants, it resulted in the eutrophication of water bodies. To better utilize the waste resources, the researchers adopted chicken manure as a raw material for biochar fabrication since it has a high amount of oxygen-containing functional groups, being an ideal candidate for biochar preparation (Qian et al., 2019).

In this study, we adopted chicken manure-derived biochar as support to load the nZVI particles for the development of nZVI/biochar composite materials. The nZVI/biochar composite materials were characterized by SEM-EDS, XRD, BET, FT-IR, XPS, magnetic properties, and zeta potential. Taking Cr(VI) as the target pollutant, batch experiments were carried out on the removal performance of the composite materials. Finally, the mechanism of nZVI@CMBC composites for Cr(VI) removal was discussed.

## 2 Materials and Methods

### 2.1 Chemicals and Materials

The chemicals, including potassium dichromate, ferrous sulfate heptahydrate (FeSO_4_∙7H_2_O), sodium borohydride (NaBH_4_), diphenyl carbamide, hydrochloric acid (HCl), acetone, absolute ethanol, sodium hydroxide (NaOH), phosphoric acid, and iron powder (Fe^0^), were purchased from Sinopharm Chemical Reagent Co., Ltd. (China). The fermented chicken manure used for biochar preparation was supplied by a local livestock plant in Fujian province, China. All the chemicals used in this study were of analytic grade. Pure water was used throughout all the experiments.

### 2.2 Preparation of Chicken Manure-Derived Biochar (CMBC)

The chicken-manure-derived biochar was synthesized by the calcination method. Specifically, the fermented chicken manure was first dried and ground into a size of 100 mesh. Afterward, a certain amount of dried chicken manure was placed in the tubular furnace for carbonization at 600°C with a heating rate of 10°C min^−1^ for 2 h in the nitrogen environment. After cooling at room temperature, the pyrolyzed chicken manure was soaked in a 0.5 M HCl solution for 24 h and then washed with pure water until the pH of the supernatant was neutral.

### 2.3 Preparation of nZVI/CMBC Composites

First, 2.5 g FeSO_4_∙7H_2_O was dissolved in a conical flask containing 100 ml water. After shaking for 30 min, 0.5 g CMBC was added to the solution and continuously shaken for 12 h. Then, the as-prepared suspension was transferred to a three-necked flask with nitrogen injection to remove the air. After 30 min, 25 ml NaBH_4_ (1.0 g/25 ml) was dropped into the suspension at the dosing rate of 2-3 ml min^−1^ (with nitrogen protection during the whole process). After the reaction was completed, the black particles were rinsed successively with deoxidized pure water and absolute ethanol three times. After freeze-drying, the nZVI/CMBC composites were obtained. In this study, different nZVI@xCMBC composites based on various mass ratios between nZVI and CMBC (i.e., x = 0.2, 0.5, 1, 2, and 5 equal to the proportion of nZVI and CMBC is 1:5, 1:2, 1:1, 2:1 and 5:1) were prepared, which were labeled as nZVI@0.2CMBC, nZVI@0.5CMBC, nZVI@1CMBC, nZVI@2CMBC, and nZVI@5CMBC, respectively.

### 2.4 Characterization

The morphology of nZVI, CMBC, and nZVI@1CMBC composites was visualized by scanning electronic microscopy (SEM) (Quanta 250, FEI, United States). Fourier Transform infrared spectroscopy (FTIR, VERTEX 70, Bruker, Germany) was employed to detect the functional groups of nZVI, CMBC, nZVI@1CMBC composites, and nZVI@1CMBC composites after Cr(VI) adsorption. X-ray diffraction (XRD, MiniFlex 600, Rigaku, Japan) was adopted to investigate the crystalline structure of nZVI, CMBC, nZVI@1CMBC composites, and nZVI@1CMBC composites after Cr(VI) adsorption. The specific surface area, porosity, and pore size distribution of nZVI, CMBC, nZVI@1CMBC composites, and Fe^0^ were measured by nitrogen adsorption using the BET analyzer (ASAP2020 HD88, Micromeritics, United States). The composition of the nZVI@1CMBC composites and ZV@1CMBC composites after Cr(VI) adsorption was determined by XPS (ESCALAB 250, Thermo Scientific, United States). The zeta potential of the nZVI@1CMBC composites was measured by using a zeta potential analyzer (NanoBrook Omni, Brookhaven, United States) at the pH range of 2.0–8.0.

### 2.5 Batch Experiments for Cr(VI) Removal

The batch reaction experiments were conducted to investigate the effect of operation parameters on Cr(VI) removal and adsorption behavior kinetics of nZVI@xCMBC composites. It is worth mentioning that the batch reaction experiments were carried out under the protection of nitrogen gas at 25°C. Before the experiments, the nitrogen gas was injected into the Cr(VI)-containing solution for 30 min to fully remove the dissolved oxygen. After the experiment, the supernatant was sampled and filtered through a porous membrane with a pore size of 0.22 μm for measurement of the Cr concentration. The removal rate of Cr(VI) by nZVI/CMBC was calculated using the following equation:

R = (C0−Ct)/C0, (1)

where R is the removal rate of Cr(VI) by nZVI/CMBC; C_t_ is the concentration of Cr(VI) in solution after t minutes; C_0_ is the initial concentration of Cr(VI) in the solution.

#### 2.5.1 Effects of the Mass Ratio of nZVI and CMBC

First, 50 ml of Cr(VI)-containing solution (50 mg L^−1^) was added to a 50 ml nut centrifuge tube, and then, 0.02 g of samples (nZVI@0.2CMBC, nZVI@0.5CMBC, nZVI@1CMBC, nZVI@2CMBC, nZVI@5CMBC, nZVI, CMBC, and Fe^0^) were added to the solution to start the reaction. After 24-h exposure and contact, the concentration of Cr(VI) in the supernatant was measured to investigate the removal performance of samples.

#### 2.5.2 Effect of the dosage of nZVI@1CMBC composites

Different dosages of the nZVI@11CMBC (0.005, 0.01, 0.02, 0.03, 0.05 and 0.1 g) were added to 50 ml Cr(VI)-containing solution (50 mg L^−1^, initial pH = 4.03). The concentration of Cr(VI) in the supernatant was determined at different exposure times (i.e., 1/12,1/6, 1/3, 1/2, 1, 3, 6, 12, 24, 48, and 72 h).

#### 2.5.3 Effect of Initial Solution pH

An amount of 0.02 g nZVI@1CMBC composite was added to the 50 ml Cr (VI) solution (50 mg L^−1^). The effect of the solution pH (2.0–10.0) on the Cr (VI) removal of the composite was explored through the determination of the concentration of Cr(VI) in the supernatant after 24-h exposure.

### 2.6 Analytical Methods

The total concentrations of Cr (Cr_total_) and Fe (Fe_total_) were measured by ICP (Optima 7000V manufactured by PerkinElmer Enterprise Management (Shanghai) Co., Ltd.). The concentration of Cr(VI) was determined by diphenyl carbamide spectrophotometry with a UV-vis spectrophotometer (Gen10S UV-Vis manufactured by Thermo Fisher Scientific Co., Ltd.) with the wavelength set at 540 nm. Cr (Ⅲ) concentration was procured by subtracting the Cr(VI) concentration from the Cr_total_ concentration.

## 3 Results and Discussion

### 3.1 Characterization of the CMBC, nZVI, and nZVI@1CMBC Composites

**FIGURE 1:**
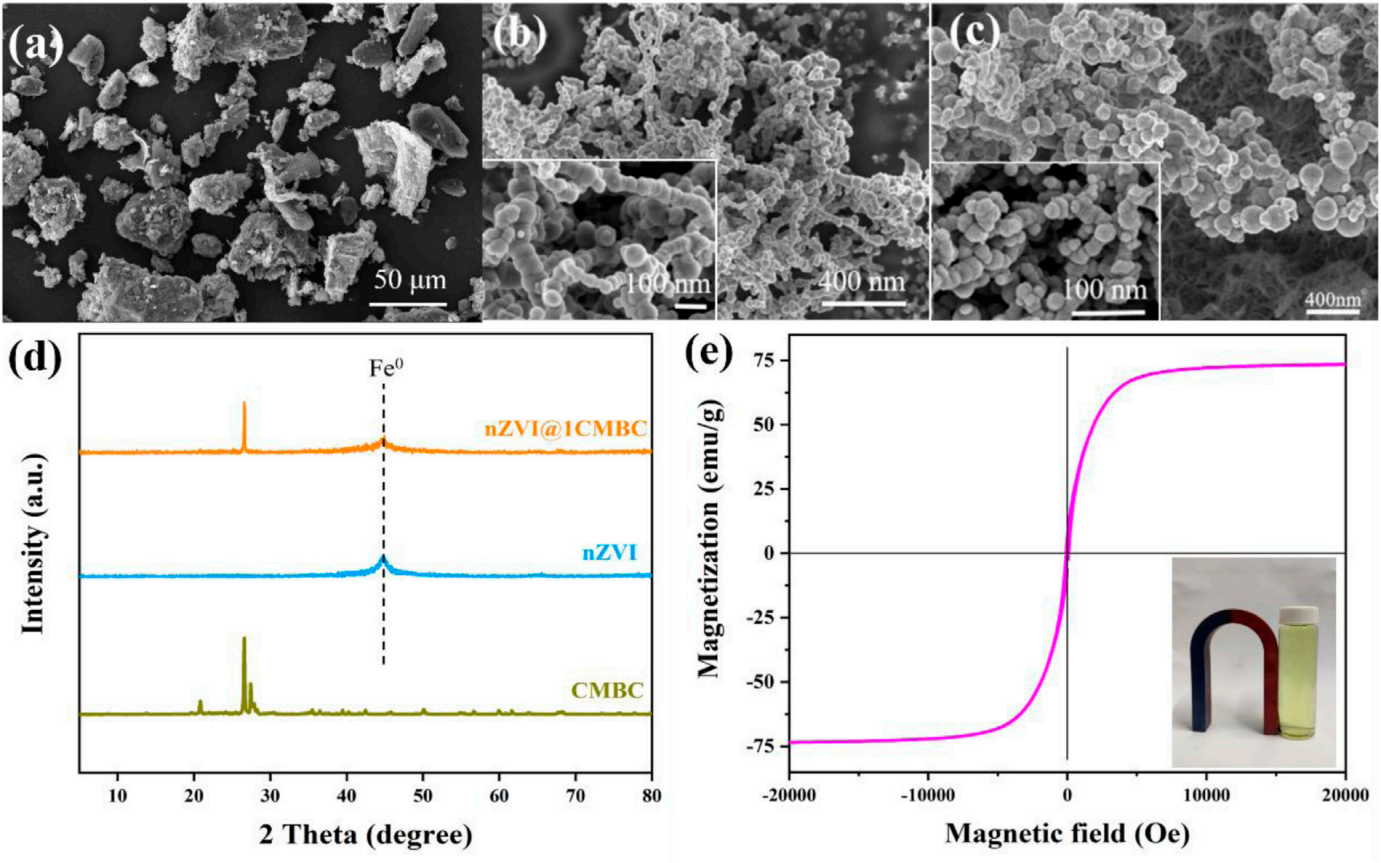
SEM images of CMBC **(A)**, nZVI **(B)**, and nZVI@1CMBC **(C)**; XRD patterns of nZVI, CMBC, nZVI@1CMBC and Cr(VI)-nZVI@1CMBC **(D)**, and magnetic hysteresis loops of nZVI@1CMBC **(E)**.

The SEM measurements of CMBC, nZVI, and nZVI@1CMBC were performed to understand their structure and morphology. As displayed in Figure 1A, the CMBC presented as a rough surface with a particle size of 10–30 μm. nZVI displayed a spherical structure with 30–90 nm diameters and tended to the chain-like structure with serious agglomeration, which was related to that typically observed in nZVI (Figure 1B). Interestingly, when nZVI is doped in CMBC, it is uniformly dispersed on the surface of CMBC, which further proved the successful synthesis of the nZVI@1CMBC composite.

XRD patterns of CMBC, nZVI, and nZVI@1CMBC are displayed in Figure 1. The CMBC had multiple diffraction peaks which can be divided into C, CaCO_3,_ and SiO_2_ (Wang et al., 2016). The main characteristic diffraction peaks 2θ = 20.85°, 26.62°, 36.53°, 45.77°, 50.11°, 63.99°, 67.70°, 68.27°, 75.61°, and 77.62° were assigned to the (100) (011), (110) (201), (112) (113), (122) (031), (302), and (220) planes of SiO_2_ (JCPDS Ref. N. 70-3,755), respectively. The peaks of 26.60°, 54.79°, 56.67°, and 60.02° were related to the (006) (012), (108), and (109) planes of C crystal (JCPDS Ref. N. 26-1076), respectively. The peaks of 35.45° and 39.49° were consistent with the 131) and (302) planes of CaCO_3_ (JCPDS Ref. N. 17-0763). XRD patterns of nZVI indicated that the peak at 26.51° was well indexed to Fe^0^ (Tian et al., 2012; Wang et al., 2014). Compared with CMBC, the XRD patterns of nZVI@1CMBC owned an additional peak of Fe^0^, implying nZVI has been successfully loaded on the CMBC.

The hysteresis curve (Figure 1E) showed that nZVI@1CMBC has the ferromagnetic property, suggesting the possibility of recycling *via* magnetic recovery. Specifically, the saturation magnetization of nZVI@1CMBC was 22.1 emu g^−1^. Furthermore, the hysteresis loop was sightless, indicating that nZVI@1CMBC is superparamagnetic and tended to scatter in the environment (Qian et al., 2019).

**FIGURE 2:**
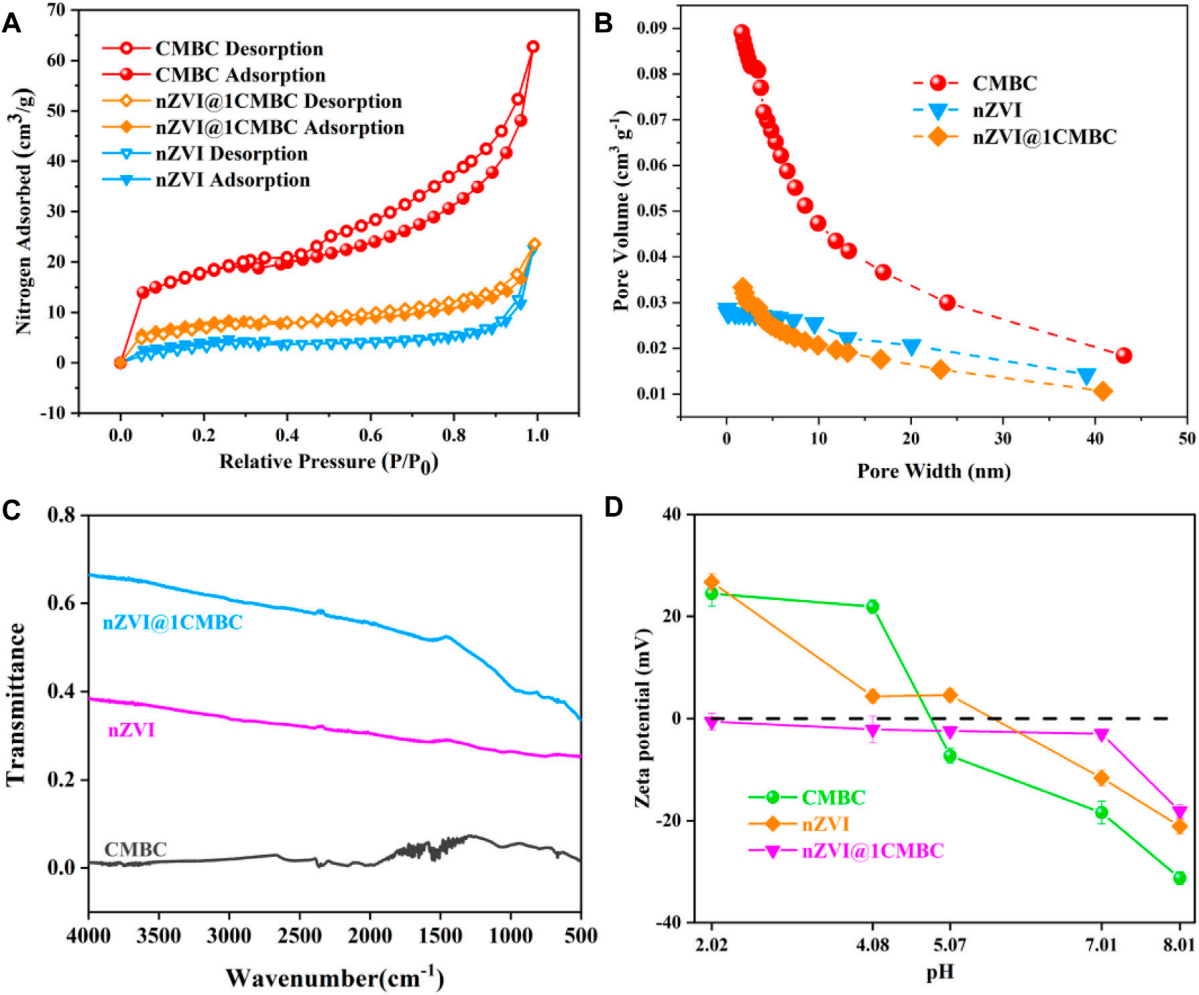
Nitrogen adsorption-desorption isotherm of nZVI, CMBC, and nZVI@1CMBC **(A)**; pore size distributions of CMBC, nZVI, and nZVI@1CMBC **(B)**; FTIR spectra of samples **(C)**; and zeta potentials of nZVI@CMBC at various pH values **(D)**.

The isotherms are displayed in Figure 2A, which reflect N_2_ adsorption–desorption measurements. In this regard, the BET surface area of nZVI, CMBC, and nZVI@1CMBC was estimated to be 15.04, 60.36, and 26.57 m^2^ g^−1^, respectively. Additionally, the aforementioned samples were classified as the type II isotherms with H4 hysteresis, according to the BDDT classification (Ruotolo and Gubulin 2011; Samuel et al., 2013). The pore volume and pore diameter of samples are displayed inFigures 2B–D and Supplementary Table S2. The nZVI@1CMBC composite had a pore volume of 0.0325 cm^3^ g^−1^ and a mean pore diameter of 2.03 nm which were between the CMBC (a pore volume of 0.0846 cm^3^ g^−1^ and a pore diameter of 2.37 nm) and nZVI (a pore volume of 0.0237 cm^3^ g^−1^ and a pore diameter of 1.15 nm), suggesting that the nZVI@1CMBC composite was successfully synthesized.

Structural changes associated with CMBC modification were explored by the FTIR analysis of CMBC, nZVI, and nZVI@1CMBC (Figure 2C). Among them, the CMBC showed broad bands in the range of 1000–1500 cm^−1^, which was due to the stretching vibration of hydroxyl groups (-OH) and carboxylic acid groups (-COOH) hydroxyl groups, and these were beneficial to the formation of complexes with metal ions (Zheng et al., 2012). Compared with the CMBC, the nZVI@1CMBC composite appeared with an absorption band at 556 cm^−1^ attributed to the Fe-O group (Wu et al., 2016), which further proved the nZVI@1CMBC composite is successfully synthesized (Xu et al., 2019). The FTIR results implied the modification of CMBC with nZVI enriched the surface functional groups of the nZVI@1CMBC composite and enhanced the adsorption of Cr(VI).

The zeta potentials of nZVI, CMBC, and nZVI@1CMBC under different pH conditions are shown in Figure 2D. It is obvious that the zeta potentials gradually decreased with the increasing pH. The zero-potential point of CMBC appeared between 4.08 and 5.07, and the zero-potential point of nZVI appeared between 5.07 and 7.01. It is worth noting that nZVI@1CMBC appeared to be electronegative at the pH range of 2.02 and 8.01, which is favorable for removing Cr(VI). The zeta results suggested that the combination of nZVI and CMBC enriches the surface electronegativity of the nZVI@1CMBC composite, which promotes the removal of Cr(VI). According to the aforementioned results, it can be inferred that the removal of Cr(VI) could be promoted by electrostatic adsorption at a pH of 2.02.

### 3.2 Advantage of nZVI@1CMBC on Cr(VI) Removal

**FIGURE 3:**
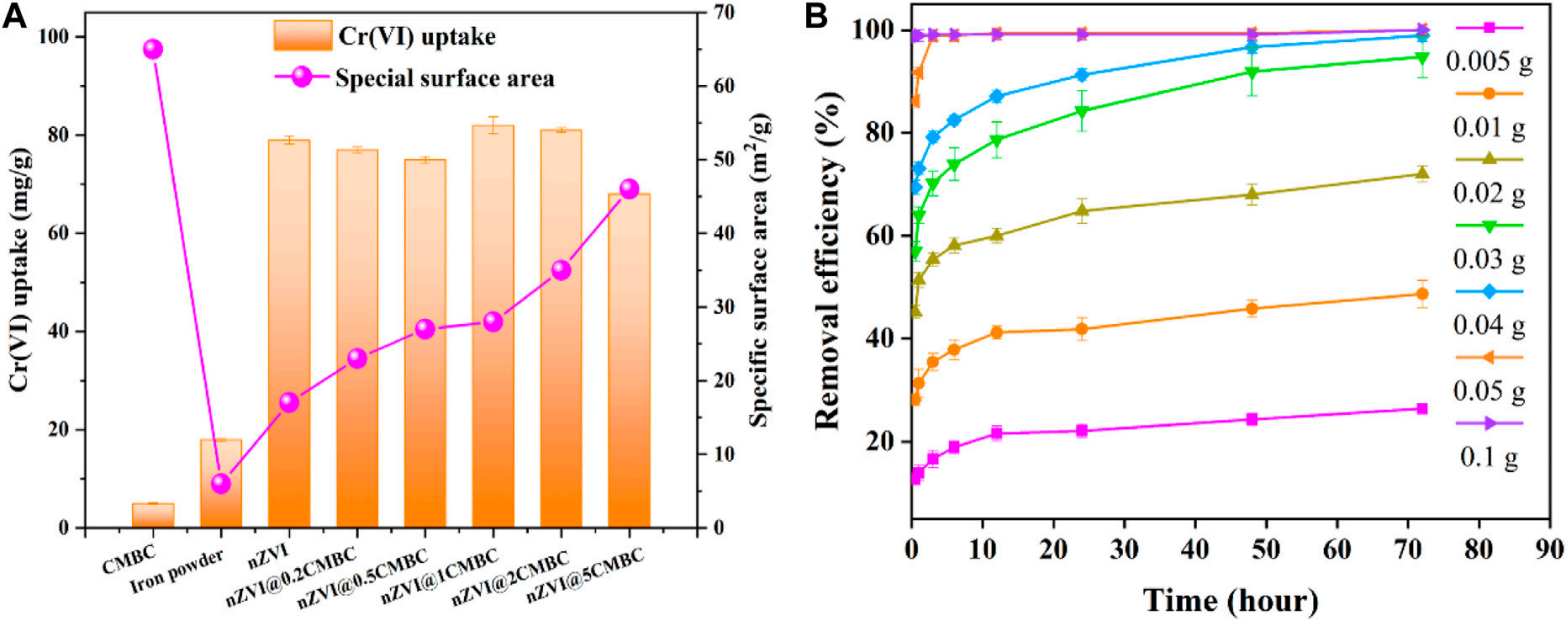
Effects of mass ratios between nZVI and CMBC on Cr(VI) removal **(A)**; effects of composite dosage on the Cr(VI) removal efficiency **(B)**.

The capability of the nZVI/CMBC composites with different mass ratios of nZVI and CMBC (nZVI@xCMBC, x = 0, 0.2, 0.5, 1, two, and 5) in removing Cr(VI) was evaluated. As shown in Figure 3A, the removal rate of Cr(VI) by nZVI@xCMBC was mainly distributed in 73.47–81.58 mg g^−1^, which was 21.8–24.1 times of CMBC (3.38 mg g^−1^) and 4.8–5.3 times of iron powder (15.27 mg g^−1^). As the proportion of CMBC increased, the surface area of the composite material increased accordingly, while the Cr(VI) removal efficiency had a little decrease, indicating that there is little correlation between the surface area and removal efficiency. When the mass ratio between nZVI and CMBC was 1, the removal rate of Cr(VI) reached 81.58 mg g^−1^, suggesting that nZVI plays an important role in removing Cr(VI). However, nZVI only experienced a removal capacity of 76.31 mg g^−1^ for Cr(VI) which is lower than nZVI@1CMBC. This is because the support of the CMBC minimizes the negative effects brought by agglomeration of nZVI, which is conducive to reducing ion leakage (Liu et al., 2013; Lv et al., 2013). Therefore, the nZVI@1CMBC composite exhibited superior performance for removing Cr(VI).

To explore the optimal dosage of the nZVI@1CMBC composite, the removal efficiency of Cr(VI) by nZVI@1CMBC was analyzed. As displayed in Figure 3D, as the dose of nZVI@1CMBC increased from 0.005 to 0.05 g, the removal efficiency of Cr(VI) increased from 16.65 to 98.92% within 72 h. However, when the dosage was 0.1 g, the removal rate of Cr(VI) only improved by 0.16% compared with the 0.05 g dosage, suggesting the optimal dosage of the nZVI@1CMBC composite to be 0.05 g (Lin et al., 2011).

### 3.3 Effects of Solution Initial pH on the Cr(VI) Removal Effect in Water

**FIGURE 4:**
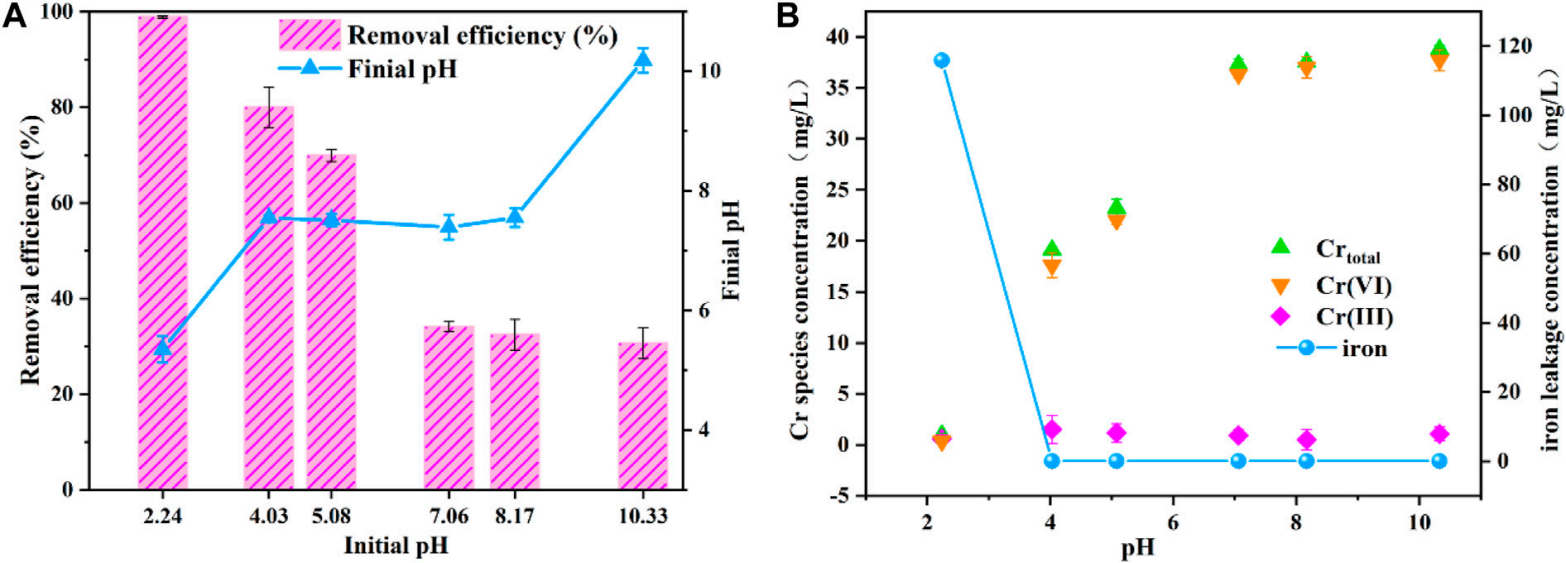
Effects of solution initial pH on the Cr(VI) removal effect in water **(A,B)**.

The removal efficiency of Cr(VI) at different pH values (2.24–10.33) was plotted in Figure 4A. As the initial pH increased, the removal efficiency of Cr(VI) apparently decreased. This can be influenced by precipitation of Fe under alkaline conditions, resulting in decreasing reduction of Cr(VI) in the solution. Based on the results obtained, when pH in the solution was 2.24, the removal efficiency reached the maximum removal efficiency (98.92%) because Fe^2+^ which existed in acidic conditions has a better ability to reduce Cr(VI) into Cr(III).

To further explore the influence of pH on the removal efficiency of Cr(VI), the contents of Cr_total_, Cr(III), Cr(VI), and iron were explored in different pH values. In Figure 4B, as the pH increased from 2.24 to 10.33, the content of Cr_total_ increased from 0.96 to 38.8 mg L^−1^, and the content of iron decreased from 115.9 to 0.07 mg L^−1^. This is due to the iron being dissolved and precipitated under the acidic conditions, and Cr(VI) was reduced to Cr(III) by Fe^0^/Fe (II) and then formed Fe-Cr precipitate (Khan et al., 2016). Moreover, when pH was 4.03, Cr (Ⅲ) separated from FeCr_2_O_4_ and existed in the form of Cr(OH)^2+^, resulting in a slight increase in the content of Cr(III). However, when the pH was between 5.08 and 10.33, stable precipitation of Cr_2_O_3_/FeCr_2_O_4_ occurred in the solution, which led to a decrease in the content of Cr (Ⅲ) (Bhaumik et al., 2012).

### 3.4 Adsorption Kinetics

**FIGURE 5:**
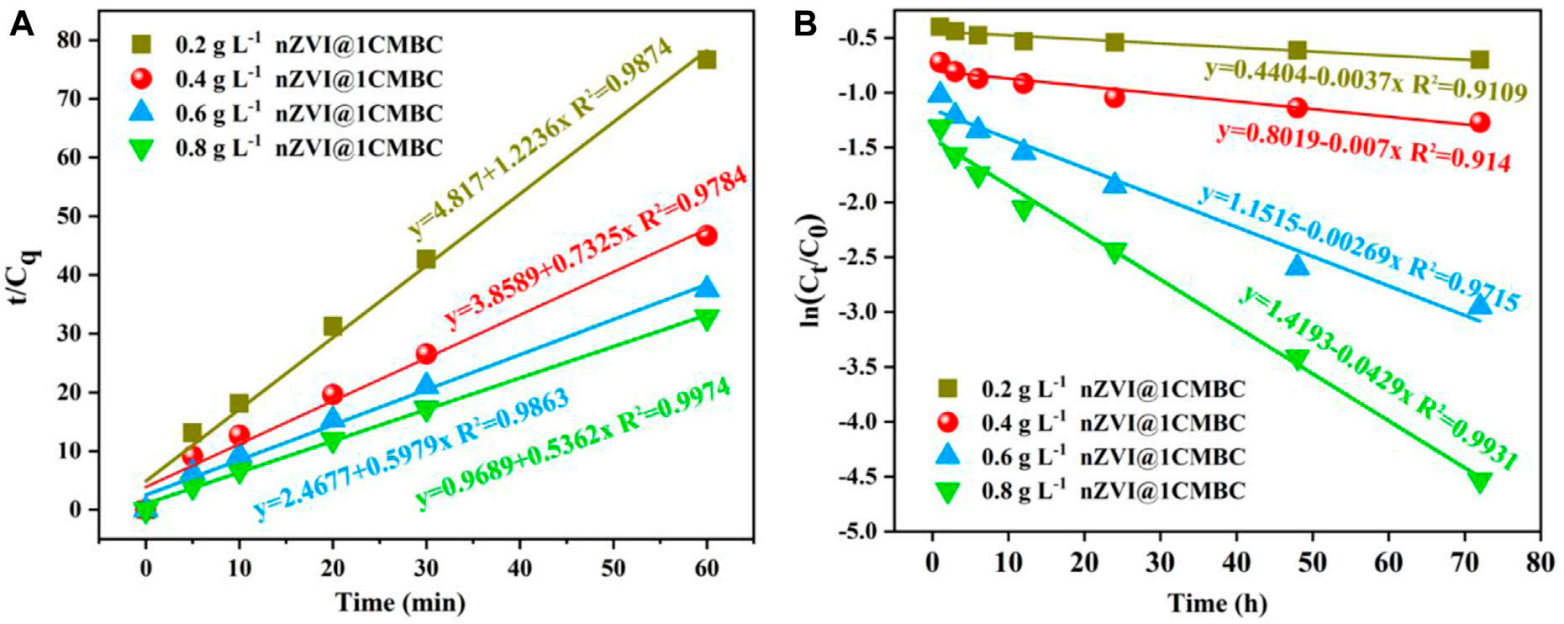
Kinetics of Cr(VI) adsorption onto nZVI@1CMBC, quasi-second order adsorption kinetics model **(A)**, and quasi-first order reaction kinetics model **(B)**.

To further elucidate the adsorption performance, the adsorption kinetics of Cr(VI) in different dosage was studied. According to the results, the reaction within 0–60 min was related to the pseudo-second-level kinetic model, and the reaction within 1–72 h was in accordance with the quasi-first order kinetic model. It means that electron transfer occurs between the nZVI@1CMBC composite and Cr(VI), suggesting that the adsorption process is chemisorption. The graphs of “t/C_q_ changing with time (C_q_ = C_0_-C_t_)", “ln (C_t_/C_0_) changing with time,” and the corresponding linear regression analysis are displayed in Figures 5A, B; Supplementary Tables S2, 3.

Within 60 min (Figure 5A), as the dosage of the nZVI@1CMBC composite increased from 0.2 to 0.8 g L^−1^, the corresponding *k*_obs_ increased from 1.2236 to 0.5362 min^−1^. However, as the dosage increased from 0.6 to 0.8 g L^−1^, *k*_obs_ had little change, implying the optimal dosage of the nZVI@1CMBC composite was 0.6 g L^−1^ (Wang et al., 2017). In the duration from 1 to 72 h (Figure 5B), the quasi-first order reaction kinetic coefficient, *k*_obs_, changed from 0.0037 to 0.0429 h^−1^, as the dosage of the composite increased from 0.2 to 0.8 g L^−1^.

### 3.5 Mechanisms of nZVI@1CMBC for Removing Cr(VI)

**FIGURE 6:**
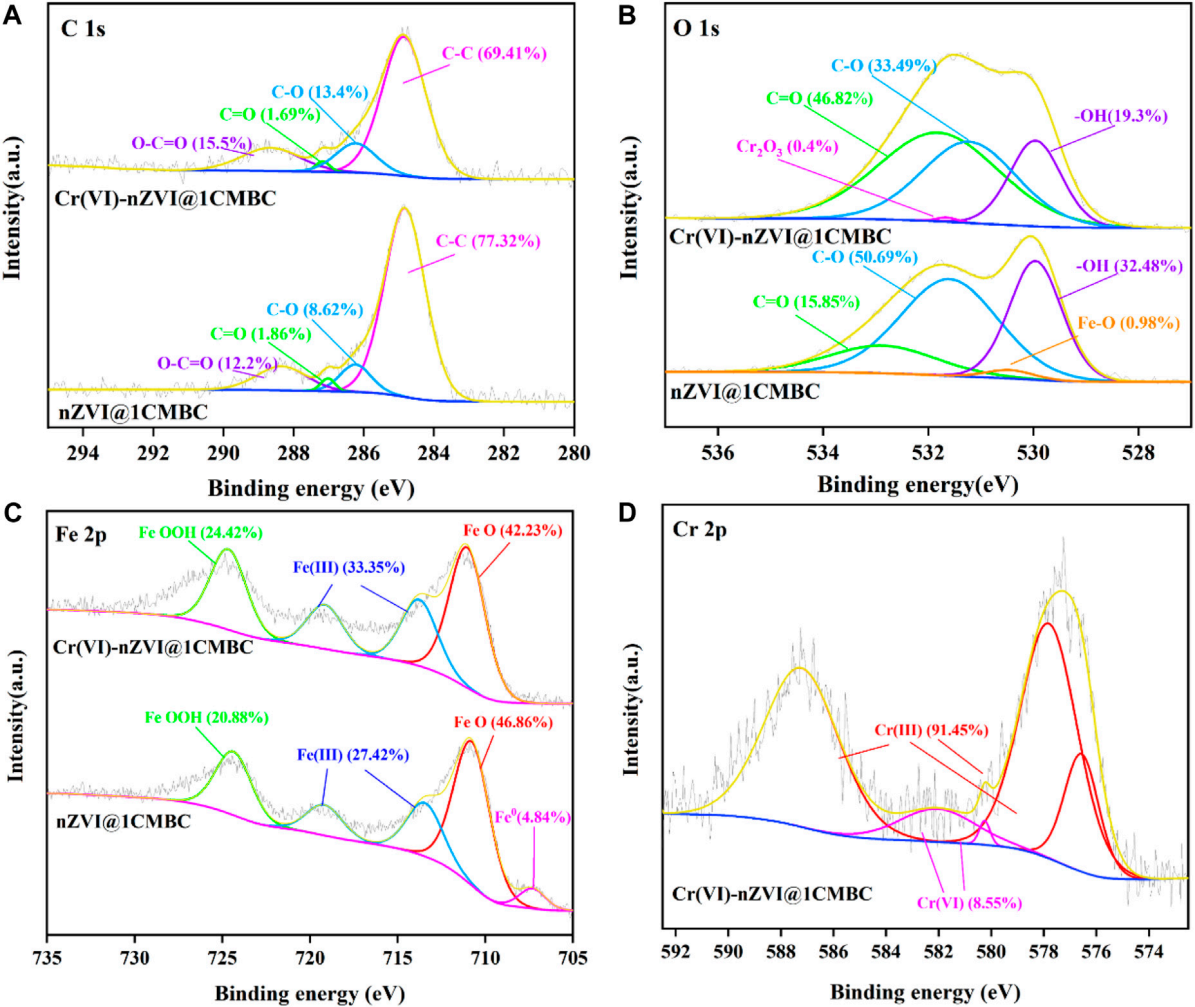
XPS spectra of nZVI@1CMBC and Cr(VI)-nZVI@1CMBC. C1s **(A)**, O1s **(B)**, Fe2p **(C)**, and Cr2p **(D)**.

To give a better understanding of the mechanism of Cr(VI) removal by nZVI@1CMBC, XPS spectra of Cr(VI)-nZVI@1CMBC before and after the reaction with Cr(VI) are demonstrated in Figure 6. For the C 1s spectrum, there were four peaks in 284.83, 286.23, 287.02, and 288.34 eV, which correspond to C-C, C-O, C=O, and O-C=O, respectively. After the reaction, the position and the content of peaks have shifted slightly, suggesting that the complexation does affect the treatment of Cr(VI). As for O 1s, after the reaction, the contents of -OH, C-O, and C=O decreased sharply, and the peak of Fe-O even disappeared. This is due to the fact that iron and its oxide were encased inside the biochar. Additionally, another peak of 531.65 eV was observed which was related to Cr_2_O_3_ (Zhang et al., 2019) due to the reduction of Cr(VI) by nZVI@1CMBC (Wang et al., 2017).

In the XPS spectra of Fe 2p, the peaks of 707.30, 710.77, 713.45, 719.17, and 724.36 eV were connected with Fe^0^, FeO, Fe (Ⅲ), and FeOOH. After the reaction, the content of FeO decreased sharply, and the peak of Fe^0^ even totally disappeared. This is probably because that Fe^0^ and Fe (Ⅱ) are oxidized by the dissolved oxygen and reacted with Cr(VI) during the reaction. Similarly, the percentages of Fe (Ⅲ) and FeOOH have increased significantly because Fe^0^ and Fe (Ⅱ) could transfer Cr(VI) to low-harmful Cr(III) with the formation of Fe(III) (Liu et al., 2013; Wu et al., 2016).

After the reaction, the Cr 2p of the Cr(VI)-nZVI@1CMBC composite was deconvoluted into five peaks. The peaks of 587.22/582.04, 580.25, 576.57, and 577.81 eV were related to Cr(III), Cr_2_O_7_^2-^, Cr_2_O_3_/Cr(OH)_3,_ and FeCr_2_O_4_, respectively (Ruotolo and Gubulin 2011). The appearance of Cr_2_O_3_/Cr(OH)_3_ and FeCr_2_O_4_ further confirms the successful reduction of Cr(VI). However, the existence of Cr_2_O_7_^2-^ indicates that a fraction of Cr(VI) is absorbed on the nZVI@1CMBC surface instead of being reduced (Tang et al., 2014; Xu et al., 2019), suggesting that the oxygen-bearing functional groups participate in the removal of Cr(VI) (Samuel et al., 2013).

Additionally, the SEM-EDS spectra of samples before and after the reaction are illustrated in Supplementary Figure S1. Compared with the nZVI@1CMBC composite, the surface of Cr-nZVI@1CMBC covered several sediments. Except for Fe, O, and C elements, Cr was detected in the EDS after the reaction. This is due to the fact that Cr_x_Fe_1-x_ (OH)_3_ and Fe_x_-Cr_y_O_4_ could be precipitated on the surface for reducing the passivation of nZVI (Karthik and Meenakshi 2015). In Supplementary Figure S2, the XRD patterns of nZVI@1CMBC and Cr(VI)-nZVI@1CMBC were analyzed. Compared with nZVI@1CMBC, Cr(VI)-nZVI@1CMBC had the peaks of 30.2°, 35.5°, and 35.6°, which were associated with FeCr_2_O_4_. The peak of Fe^0^ in Cr(VI)-nZVI@1CMBC had decreased completely which was consistent with the XPS. The FTIR spectrum of Cr(VI)-nZVI@1CMBC is shown in Supplementary Figure S3. After the reaction, the peaks of 1320, 1060, and 465 cm^−1^ have vanished completely, and the peak of 980 cm^−1^ which corresponded to Cr-O groups has appeared (Tian et al., 2012). Zeta potentials of nZVI@1CMBC and Cr(VI)-nZVI@1CMBC are demonstrated in Supplementary Figure S4. The surface electrical charge of Cr-nZVI@CMBC was slightly higher than that of the nZVI@1CMBC composite, which was due to the formation of Cr (Ⅲ) on the surface. In summary, these results demonstrated that absorption and complexation effects are presented in the removal of Cr(VI).

**FIGURE 7:**
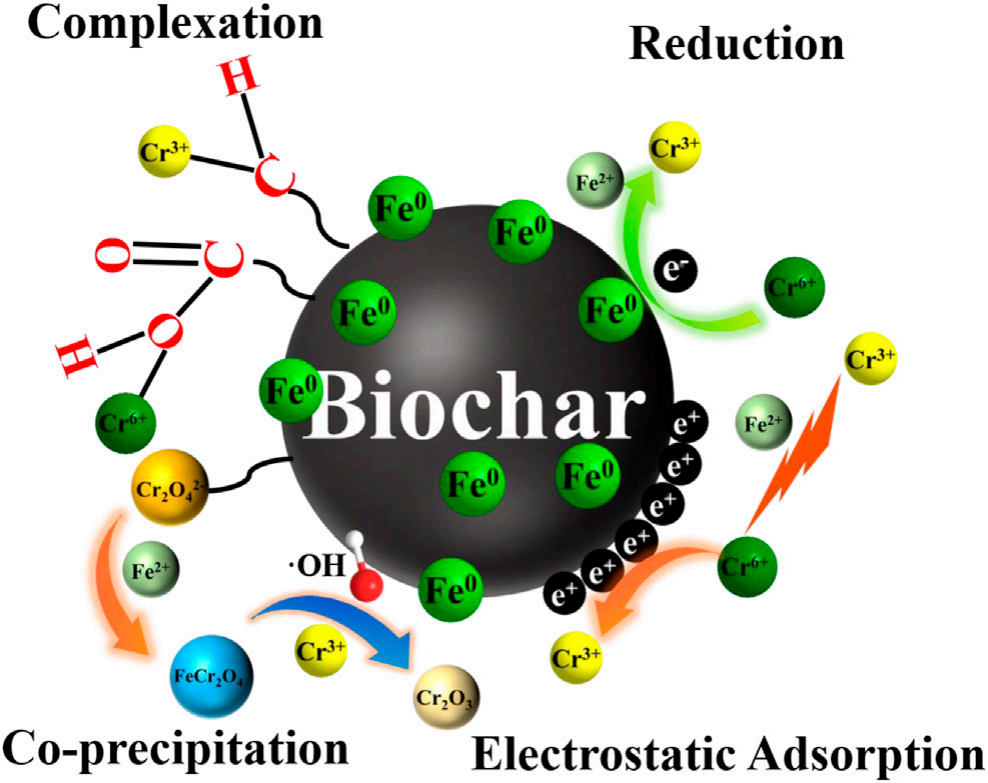
Illustration of the theoretical Cr(VI) removal mechanisms of nZVI@CMBC, including reduction, electrostatic attraction, surface complexation, and co-precipitation.

Based on the abovementioned discussion, it is assumed that Cr(VI) was removed by adsorption, reduction, and complexation (Figure 7). The potential reaction processes are demonstrated as follows: 1) the excellent pores and oxygen-bearing functional groups of nZVI@1CMBC (C-O, C=O, -OH, and O-C=O) had an adsorption effect on Cr(VI); 2) electrostatic adsorption was beneficial to the removal of Cr(VI); 3) under acidic conditions, parts of Fe^0^ and Cr(VI) could react with H^+^ and translate into Fe (Ⅱ) and Cr (Ⅲ) (Eqs. 3, 4); 4) Cr(VI) could obtain electrons from Fe^0^/Fe(II) and transfer them to Cr(III) (Eqs. 4, 5); 5) Forming Fe (Ⅲ)-Cr (Ⅲ) co-precipitation is *via* ion exchange (Eqs. 6–8; 6) the excellent conductivity of biochar in the nZVI@1CMBC composite also plays a significant role in accelerating electron transfer (Ruotolo and Gubulin 2011; Lv et al., 2013; Tang et al., 2014).

$${\text{Fe}}^{\text{0}}{+2\text{H}}^{+}\to {\text{Fe}}^{2+}{+\text{H}}_{2},$$ (2)

$${\text{HCrO}}_{4}^{-}{+7\text{H}}^{+}{+3\text{e}}^{-}↔{\text{Cr}}^{3+}{+4\text{H}}_{2}\text{O,}$$ (3)

$${3\text{Fe}}^{\text{0}}{+\text{Cr}}_{2}{\text{O}}_{7}^{2-}{+14\text{H}}^{+}\to {3\text{Fe}}^{2+}{+2\text{Cr}}^{3+}{+7\text{H}}_{2}\text{O,}$$ (4)

$${3\text{Fe}}^{2+}{+\text{HCrO}}_{4}^{-}{+7\text{H}}^{+}\to {3\text{Fe}}^{3+}{+\text{Cr}}^{3+}{+4\text{H}}_{2}\text{O,}$$ (5)

$${(1-\text{x})\text{Fe}}^{3+}{+\text{xCr}}^{3+}{+3\text{H}}_{2}\text{O}\to {(\text{Cr}}_{\text{x}}{\text{Fe}}_{1-\text{x}}{)(\text{OH})}_{3}{(\text{s})+3\text{H}}^{+},$$ (6)

$${2\text{Cr}}^{3+}{+6\text{OH}}^{-}↔{2\text{Cr}\left(\text{OH}\right)}_{3}\left(\text{s}\right)↔{\text{Cr}}_{2}{\text{O}}_{3}{\left(\text{s}\right)+3\text{H}}_{2}\text{O,}$$ (7)

$${\text{Fe}}^{2+}{+\text{Cr}}_{2}{\text{O}}_{4}^{2-}↔{\text{FeCr}}_{2}{\text{O}}_{4}\left(\text{s}\right)\text{.}$$ (8)

Meanwhile, the contribution rates of various mechanisms in the reaction are displayed in Table 1, and different contents of Cr_total_, Cr(III), and Cr(VI) were calculated. According to the results, the content of Cr(VI), which was reduced by nZVI@1CMBC, accounted for 91.98% and the rest of that only accounted for 8.02%.

**TABLE 1:** Removal Cr(VI) and suspended component by nZVI@1CMBC.

Item	Cr(VI)	Cr(III)	Cr_total_
Equilibrium concentration (mg L^−1^)	17.60	1.51	19.11
Content of Cr in the equilibrium solution (mg)	0.88	0.08	0.96
Percentage of Cr after reaction (%)	8.55%	91.45%	100%
Content of Cr after the reaction (mg)	0.13	1.41	1.54

## 4 Conclusion

In this study, the nZVI@1CMBC composite was developed by combining the advantages of zero-valent iron (nZVI) nanoparticles and chicken manure biochar (CMBC). nZVI@1CMBC exhibited better adsorption capacity for removing Cr(VI) than nZVI and CMBC, and the removal efficiency could reach 98.92% within 72 h. The results indicate that the high specific surface area and the oxygen-containing functional groups (-COOH, -OH, O-C=O) detected on the surface benefit from the removal of Cr(VI) *via* the synergistic effect of reduction, electrostatic adsorption, and surface complexation. Compared with other traditional removal agents, nZVI@1CMBC is cost-effective and favors neutralizing the pH of wastewater containing Cr(VI). Moreover, due to the strong magnetic properties, the nZVI@1CMBC composite can be effectively recovered for reuse. This study provides an important and useful concept for the design of the new biochar-based composites for the effective removal of Cr (VI).

## Data Availability

The original contributions presented in the study are included in the article/Supplementary Material; further inquiries can be directed to the corresponding author.
