# Supplementary material for: Removal of Chromium (VI) by a Magnetic Nanoscale Zerovalent Iron–Assisted Chicken Manure-Derived Biochar: Adsorption Behavior and Synergetic Mechanism
Source: Front Bioeng Biotechnol. 2022 Jul 6;10:935525. doi: 10.3389/fbioe.2022.935525 (PMC9298784; doi:10.3389/fbioe.2022.935525)
Supplement: Supplementary file 1 [file DataSheet1.docx]

**Supporting Information**

**Removal of chromium (VI) by a magnetic nanoscale Zerovalent Iron assisted chicken manure-derived biochar: Adsorption behavior and synergetic mechanism**

Shengqiong Fang^a^, Xiaoyi Huang^a^, Shuangling Xie^a^, Jiale Du^a^, Jianlong Zhu^a^, Kai Wang^a^, Qinglin Zhuang^a^, Xuan Huang^b,^*

*Corresponding author: shian25.huang@foxmail.com

^a^ College of Environment and Safety Engineering, Fuzhou University, 350108 Fuzhou, China

^b^ Jiangsu DDBS Environmental Remediation Co., Ltd., 210012 Nanjing, China

**Content**

**Fig. S1.** EDS-SEM image of nZVI@CMBC and Cr-nZVI@CMBC.

**Fig. S2.** XRD of nZVI@1CMBC and Cr(VI)-nZVI@1CMBC.

**Fig. S3.** FTIR of nZVI@1CMBC and Cr(VI)-nZVI@1CMBC.

**Fig. S4.** zeta potentials of nZVI@1CMBC and Cr(VI)-nZVI@1CMBC.

**Table S1.** Specific surface area, pore size, and pore volume of the samples.

**Table S2.** Pseudo first-order, used for simulating Cr(VI) sorption kinetic data and the corresponding fitting parameters.

**Table S3.** Parameters of pseudo-second-order equation for adsorption.


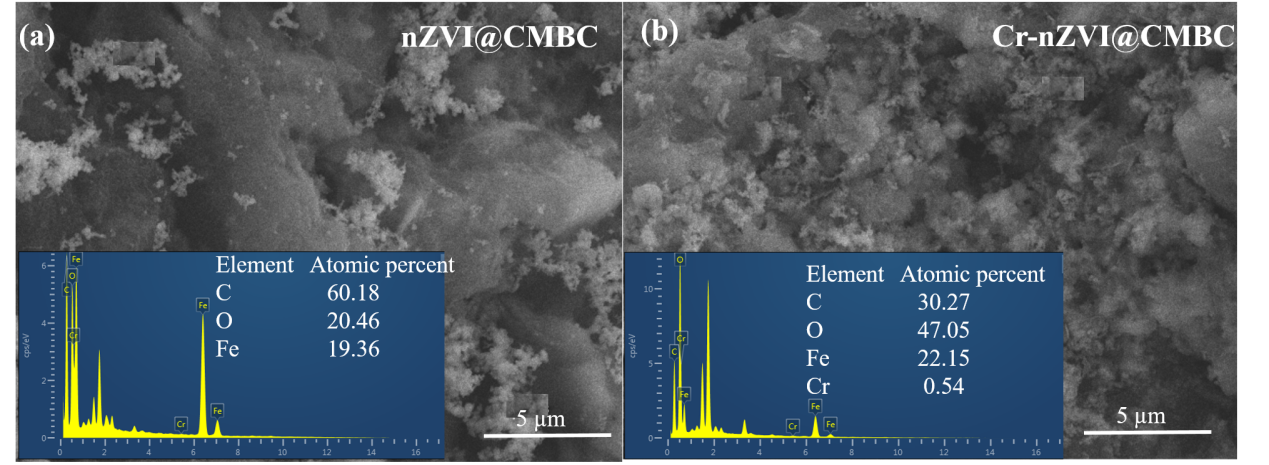


**Fig. S1.** EDS-SEM image of nZVI@CMBC(a) and Cr-nZVI@CMBC(b).


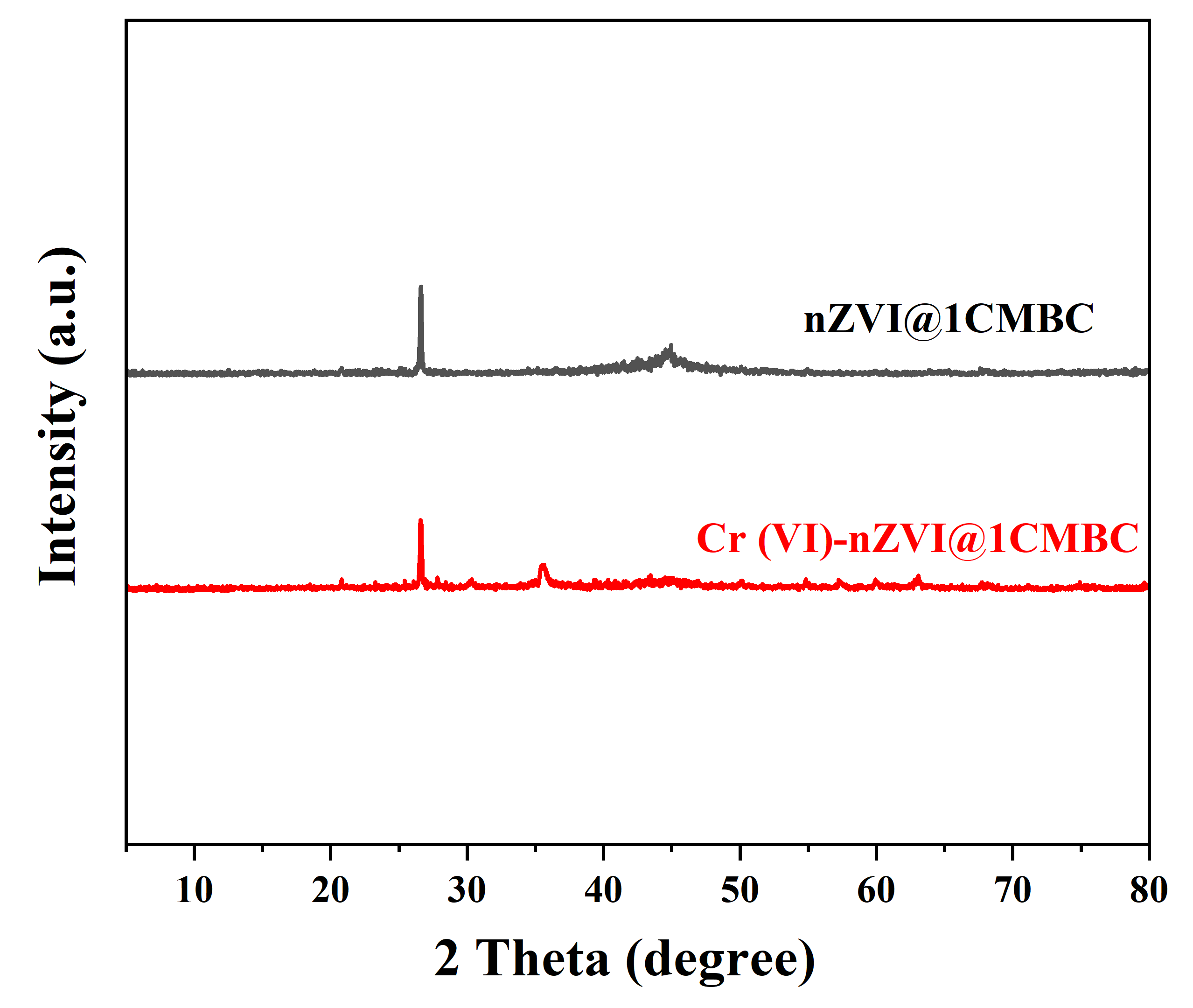


**Fig. S2.** XRD of nZVI@1CMBC and Cr(VI)- nZVI@1CMBC


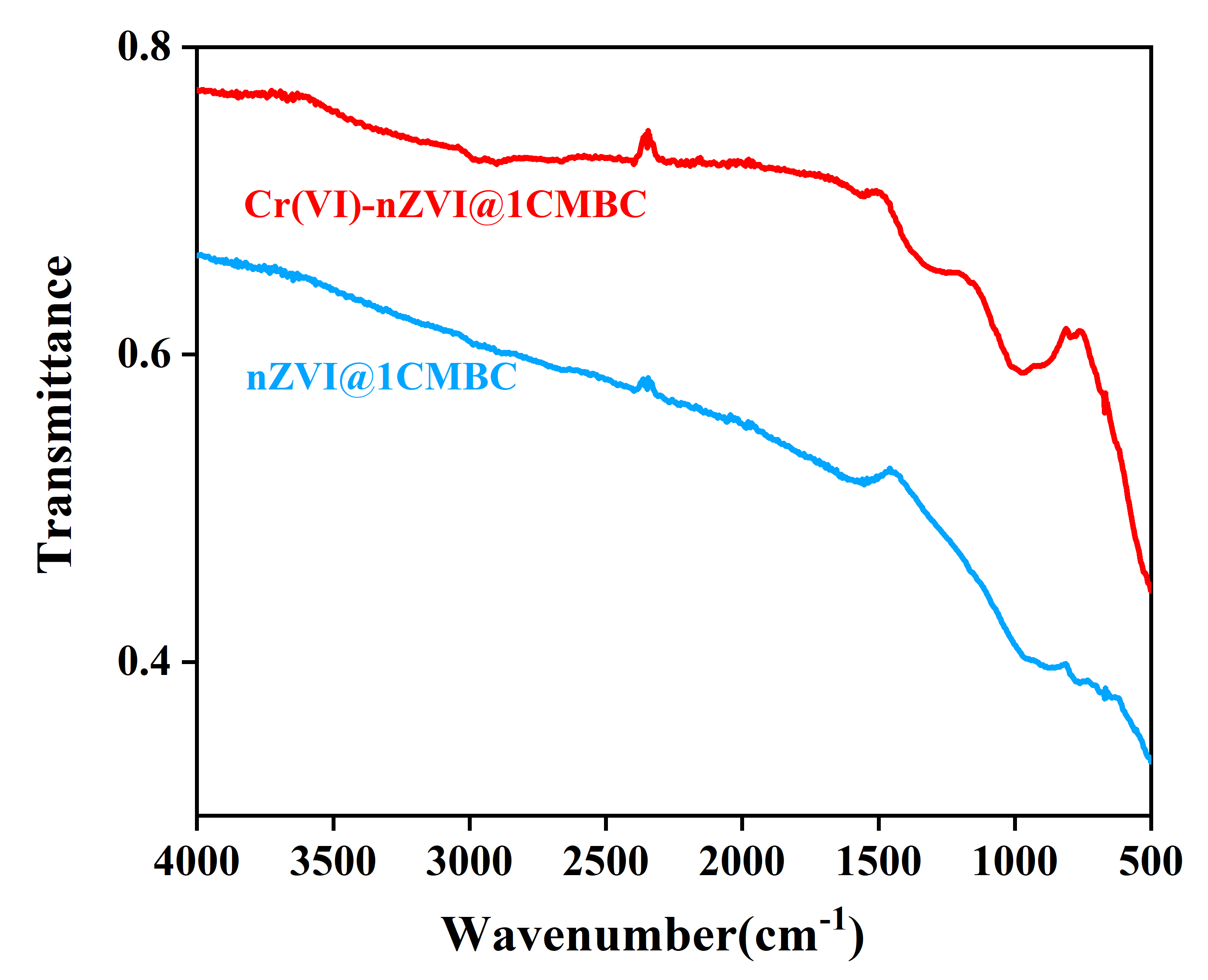


**Fig. S3.** FTIR of nZVI@1CMBC and Cr(VI)- nZVI@1CMBC


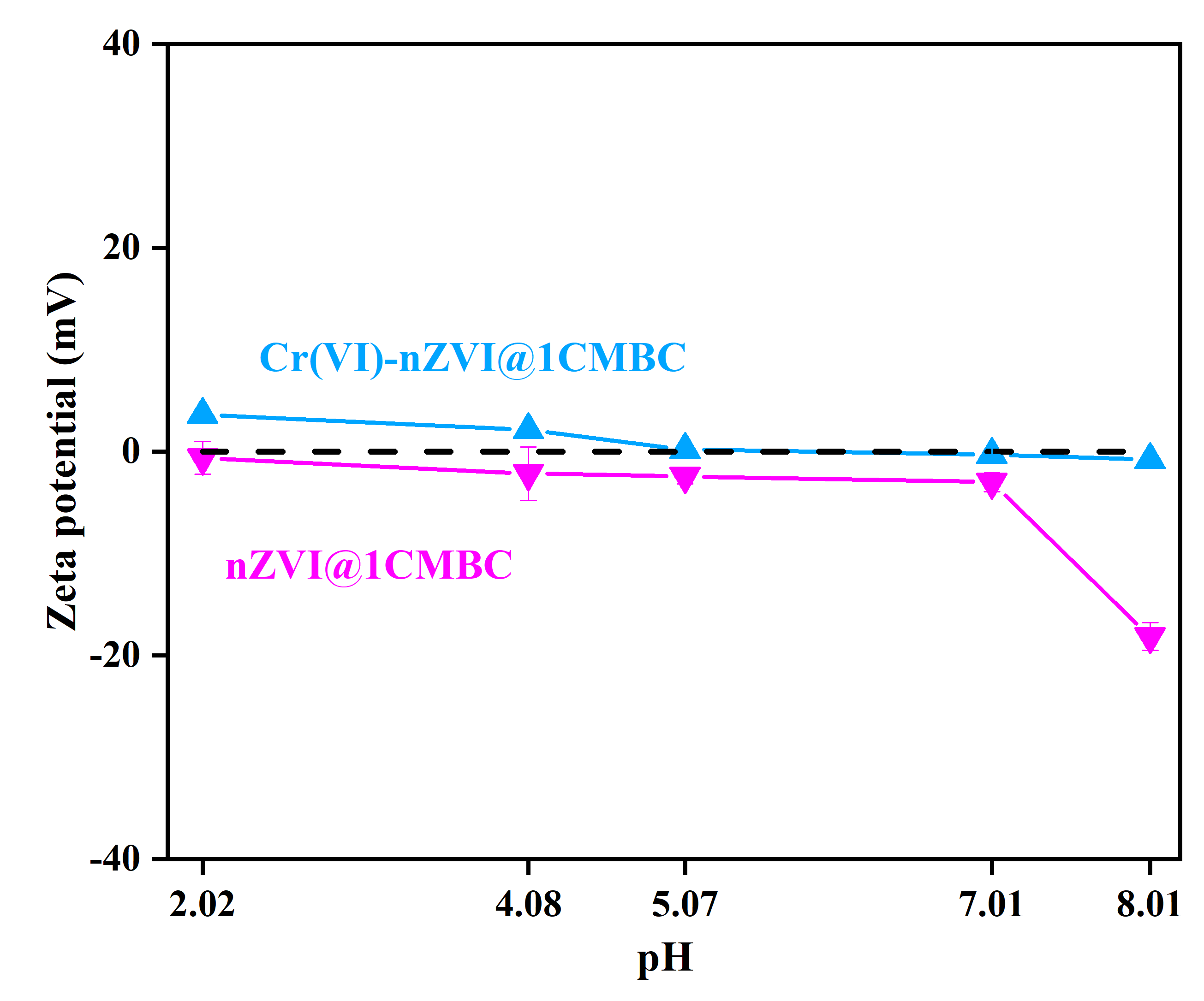


**Fig. S4.** Zeta potentials of nZVI@1CMBC and Cr(VI)- nZVI@1CMBC

**Table S1** Specific surface area, pore size, and pore volume of the samples.

| Sample | BET SurfaceArea  (m^2^ g^-1^) | Total PoreVolume  (cm^3^ g^-1^) | Pore diameter  (nm) |
| --- | --- | --- | --- |
| nZVI | 15.04 | 0.0350 | 9.31 |
| CMBC | 60.36 | 0.0970 | 6.43 |
| nZVI@0.2CMBC | 16.25 | 0.0494 | 9.31 |
| nZVI@0.5CMBC | 17.63 | 0.0265 | 6.01 |
| nZVI@1CMBC | 26.57 | 0.0365 | 5.49 |
| nZVI@2CMBC | 34.52 | 0.0572 | 6.62 |
| nZVI@5CMBC | 45.85 | 0.0669 | 5.84 |
| Fe^0^ | 5.74 | 0.0004 | 0.24 |

**Table S2** Parameters of pseudo-first-order equation for adsorption

| nZVI@1CMBC（g） | Pseudo-first-order model | R^2^ |
| --- | --- | --- |
| 0.01 | $\text{y}=\text{-}0.4404\text{-}0.0037x$ | 0.9109 |
| 0.02 | $\text{y}=\text{-}0.8019\text{-}0.007x$ | 0.9145 |
| 0.03 | $\text{y}=\text{-}1.1515\text{-}0.0269x$ | 0.9715 |
| 0.04 | $\text{y}=\text{-}1.4193\text{-}0.0429x$ | 0.9931 |

**Table S3** Parameters of pseudo-second-order equation for adsorption

| nZVI@1CMBC（g） | Pseudo-second-order model | R^2^ |
| --- | --- | --- |
| 0.01 | $\text{y}=4.817+1.2236x$ | 0.9874 |
| 0.02 | $\text{y}=3.8589+0.7325x$ | 0.9784 |
| 0.03 | $\text{y}=2.4677+0.5979x$ | 0.9863 |
| 0.04 | $\text{y}=0.9689+0.5362x$ | 0.9974 |
